# Synthesis and Optimization of Mesoporous Silica Nanoparticles for Ruthenium Polypyridyl Drug Delivery

**DOI:** 10.3390/pharmaceutics13020150

**Published:** 2021-01-24

**Authors:** Siti Norain Harun, Haslina Ahmad, Hong Ngee Lim, Suet Lin Chia, Martin R. Gill

**Affiliations:** 1 Department of Chemistry, Faculty of Science, Universiti Putra Malaysia, Serdang 43400 UPM, Selangor, Malaysia; norain.harun@gmail.com (S.N.H.); hongngee@upm.edu.my (H.N.L.); 2 UPM-MAKNA Cancer Research Laboratory, Institute of Bioscience, Unversiti Putra Malaysia, Serdang 43400 UPM, Selangor, Malaysia; suetlin@upm.edu.my; 3 Department of Microbiology, Faculty of Biotechnology and Biomolecular Sciences, Universiti Putra Malaysia, Serdang 43400 UPM, Selangor, Malaysia; 4 Department of Chemistry, Swansea University, Swansea SA2 8PP, UK; m.r.gill@swansea.ac.uk

**Keywords:** mesoporous silica nanoparticles, ruthenium polypyridyl, drug delivery, IC_50_

## Abstract

The ruthenium polypyridyl complex [Ru(dppz)_2_PIP]^2+^ (dppz: dipyridophenazine, PIP: (2-(phenyl)-imidazo[4,5-f ][1,10]phenanthroline), or Ru-PIP, is a potential anticancer drug that acts by inhibiting DNA replication. Due to the poor dissolution of Ru-PIP in aqueous media, a drug delivery agent would be a useful approach to overcome its limited bioavailability. Mesoporous silica nanoparticles (MSNs) were synthesized via a co-condensation method by using a phenanthrolinium salt with a 16 carbon length chain (Phen-C_16_) as the template. Optimization of the synthesis conditions by Box–Behnken design (BBD) generated MSNs with high surface area response at 833.9 m^2^g^−1^. Ru-PIP was effectively entrapped in MSNs at 18.84%. Drug release profile analysis showed that Ru-PIP is gradually released, with a cumulative release percentage of approximately 50% at 72 h. The release kinetic profile implied that Ru-PIP was released from MSN by diffusion. The in vitro cytotoxicity of Ru-PIP, both free and MSN-encapsulated, was studied in Hela, A549, and T24 cancer cell lines. While treatment of Ru-PIP alone is moderately cytotoxic, encapsulated Ru-PIP exerted significant cytotoxicity upon all the cell lines, with half maximal inhibitory concentration (IC_50_) values determined by MTT (([3-(4,5-dimethylthiazol-2-yl)-2,5-dephenyltetrazolium bromide]) assay at 48 h exposure substantially decreasing from >30 µM to <10 µM as a result of MSN encapsulation. The mechanistic potential of cytotoxicity on cell cycle distribution showed an increase in G1/S phase populations in all three cell lines. The findings indicate that MSN is an ideal drug delivery agent, as it is able to sustainably release Ru-PIP by diffusion in a prolonged treatment period.

## 1. Introduction

In the development of therapeutic drugs, ruthenium-based complexes offer stable and predictable structures prepared through reliable routes, multiple ligand functionalization in improving biomolecular binding selectivity, and well-explored knowledge of the resultant biological effects [1,2,3]. While many ruthenium complexes have been developed, potent therapeutic compounds also face challenges. Many ruthenium complexes developed for therapy are hydrophobic, which makes it problematic to prepare a higher concentration of the complex in a small volume of aqueous media [4]. Although hydrophilicity can be improved, this often comes at a cost of reduced bioactivity. Instead of modifying the chemical properties of the complexes, which could alter therapeutic potency, an alternative solution is to introduce a suitable drug delivery system. Various delivery strategies have been developed for the purpose of improving the performance of ruthenium complexes [5].

The presence of silica is extensive in living nature [6], reflecting its biocompatibility [7]. The discovery of mesoporous silica nanoparticles (MSNs) was made by Exxon-Mobil scientists in 1942 [8,9]. MSNs possess unique characteristics, such as high surface area, tunable pore and particle size, and facile surface functionalization [10,11]. Mesoporous silica nanoparticles possess abundant silanol groups with a natural affinity for phospholipids that promotes their uptake by living cells via endocytosis [12]. MSN with a pore size ranging from 2 to 5 nm are excellent candidates for drug delivery, and mesoporous porosity is tunable to the type and size of drugs [13]. The non-polar cavities of MSN are suitable for hydrophobic ruthenium drugs, and the mesoporous channel can preserve the drug in a non-crystalline state, which facilitates drug dissolution [14]. The inert properties of MSNs are not predicted to hinder the therapeutic effect of ruthenium complex. The biosafety results of the cytotoxicity, biodegradation, biodistribution, and excretion of MSNs in vivo have been satisfactory [15,16].

Substantial research has been conducted in order to find the most effective way to synthesize MSNs with tailored morphologies for the desired application [17]. A general method to produce MSNs is by using a surfactant as a template, around which the formation of silica network would occur [18]. Through this method, the selection of the template provides a way to control the morphology, therefore offering different properties of the produced materials. Researchers have discovered that the concentration of the template affects the colloidal state of MSNs, and also the thickness of external silica layer of the particles [19,20]. Surfactants, such as quaternary ammonium salts, are often used as templates guiding the assembly of silica precursors to form silica particles. Common agents used to provide basic conditions for the reaction are ammonia, sodium hydroxide (NaOH), and triethanolamine (TEA). Reactions utilizing ammonia and NaOH often require much more diluted conditions, which may lead to difficulty in collecting the product, while the reaction using TEA often requires smaller volumes [21]. 

Response surface methodology (RSM) is a collective statistical and mathematical method employed for modeling and analyzing production problems. The main objective of this technique is to statistically explore the relationship of several variables and one or more responses. The Box–Behnken design (BBD) is a class of second-order designs based on three-level, interlocking, incomplete factorial designs [22]. BBD provides relatively fewer design points than other full factorial designs for determining a complex response function, which is experimentally more efficient and less expensive [23]. In addition, all design points in the BBD stay within the safe operating zone, as the design does not include combination of variables at their highest or lowest level simultaneously, thus avoiding experiments under extreme conditions [24].

**Figure 1:**
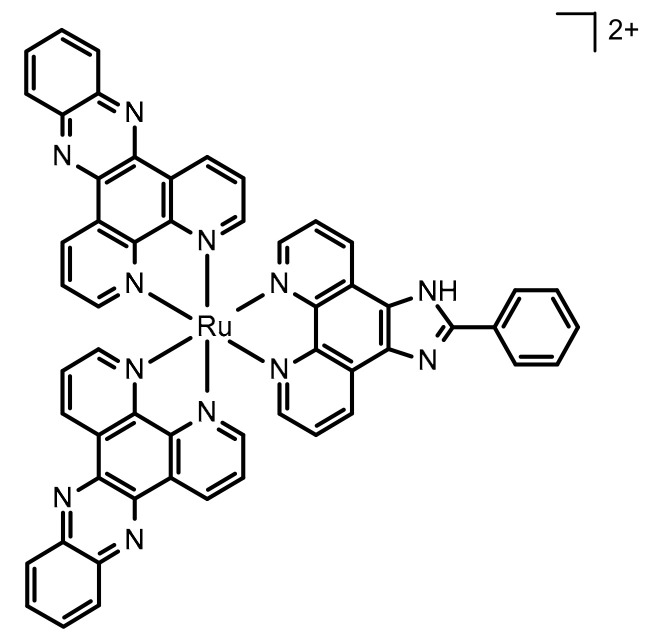
Chemical structure of Ru-PIP ([Ru(dppz)_2_PIP]^2+^ (dppz: dipyridophenazine, PIP: (2-(phenyl)-imidazo[4,5-f ][1,10]phenanthroline)).

Previous work has shown that the DNA metallo-intercalator [Ru(dppz)_2_PIP]^2+^ (dppz: dipyridophenazine, PIP: (2-(phenyl)-imidazo[4,5-f ][1,10]phenanthroline; Figure 1), or Ru-PIP, binds DNA with high affinity and inhibits the proliferation of cancer cells by stalling DNA replication forks [25,26]. However, the complex suffers from both poor aqueous solubility and relatively mild potency. The aim of the present study was to optimize the surface area of MSNs by manipulating synthesis conditions using RSM and to utilize the optimized MSN as a drug delivery system for Ru-PIP. Due to the polypyridyl chemistry of Ru-PIP, phenanthrolinium salt was used as an alternative template to form MSNs through the co-condensation method, and BBD was employed to investigate the reaction parameters. Drug release and the kinetic profile of Ru-PIP from MSNs were evaluated, while the in vitro cytotoxicity of MSN-delivered Ru-PIP was investigated on several cancer cell lines, including cervical Hela, lung A549, and bladder T24. 

## 2. Materials and Methods 

### 2.1. Materials and Reagents

For this process, 1,10-phenanthroline (Sigma Aldrich, St. Louis, MO, USA), 1-bromohexadecane (Acros Organic, Geel, Belgium), acetonitrile (Merck, Kenilworth, NJ, USA), diethyl ether (Fisher Scientific, Waltham, MA, USA), tetraethylorthosilicate (TEOS) (Merck, Kenilworth, NJ, USA), triethanolamine (TEA) (Merck, Kenilworth, NJ, USA), absolute ethanol (J. Kollins, UK), and phosphate buffer saline (PBS) (Sigma Aldrich, St. Louis, MO, USA) were acquired. All chemicals and materials were of analytical grade and purchased from Sigma Aldrich (St. Louis, MO, USA), Thermo Fisher (Waltham, MA, USA), Acros Organic (Geel, Belgium), Merck (Kenilworth, NJ, USA), or J. Kollins (United Kingdom); they were used without any further purification unless specified otherwise. The [Ru(dppz)_2_PIP]^2+^ was prepared according to a previously reported method [25]. 

Normal human dermal fibroblasts (NHDFs), as well as the Hela, A549, and T24 cell lines, were obtained from Department of Microbiology, Faculty of Biotechnology and Biomolecular Sciences, Universiti Putra Malaysia, Malaysia. NHDFs were cultured in Dulbecco’s modified Eagle’s medium (DMEM) with 5% fetal bovine serum (FBS), and all cancer cell lines (Hela, A549, and T24) were cultured in DMEM with 10% FBS.

### 2.2. Synthesis of Mesoporous Silica Nanoparticles

The template, a phenanthrolinium salt with 16-carbon chain length, was prepared by following a typical synthesis of quartenium salts described in previously reported procedures, with some modifications [27,28,29,30]. Phen-C_16_ (Figure S1) was obtained by reacting 16 mmol 1,10-phenanthroline dissolved in 5 mL of ethanol with 25 mmol 1-bromohexadecane. The mixture was stirred and refluxed at 170 °C for 24 h. The salt was purified by dissolving the crude in a minimal amount of acetonitrile and precipitated with ether. The final product was collected through filtration and dried in a desiccator to remove any moisture. Phen-C_16_: *m*/*z* = [M^+^] 405; ^1^H-NMR (500 MHz, DMSO-d6, δ): 9.65 d, 9.42 d, 9.29 dd, 8.83 dd, 8.44 m, 8.09 m, 5.89 dd, 2.51 dd, 2.06 m, 1.52 m, 1.24 m, and 0.83 dd (d; doublet, dd; doublet of doublet and m; multiplet).

The mesoporous silica nanoparticles (MSNs) were prepared by following a typical synthesis process [31,32] with a cationic surfactant. A calculated mass of 1-hexadecyl-1,10-phenanthrolinium bromide was dissolved in 20 mL of deionized water. An appropriate amount of triethanolamine was added, and the solution was stirred at 550 rpm at a certain temperature for 30 min. Then, 1.5 mL of tetraethyl orthosilicate was added dropwise, and the reaction time was prolonged for 2 h. The particles were isolated with centrifugation and washed with ethanol several times. The phenanthrolinium template was removed by stirring the as-synthesized particles in a mixed solution of 30.0 mL ethanol and 1.0 mL hydrochloric acid (38%) for 8 h. The MSNs were isolated by centrifugation and washed with excess ethanol to remove the phenanthrolinium salt residue. The same process was repeated in order to remove the template completely. The obtained solid was dried in an oven at 50 °C for 24 h.

### 2.3. Experimental Design

MSNs with different particle sizes were synthesized by controlling the reaction temperature, amount of triethanolamine (TEA) and mass of template used. A Box–Behnken design was employed to study the effects of variable parameter interactions on the morphology of MSNs. The process variables for this study are listed in Table 1. The levels were selected based on preliminary experiment trials and literature reviews. The corresponding BET isotherm linear plot is listed in Figure S3.

### 2.4. In Vitro Drug Loading and Release

The drug solution was prepared by dissolving Ru-PIP in ethanol with a concentration of 1 mM. Then, 50 mg of MSN were dispersed in the drug solutions, followed by 1 h of sonication. The solutions were stirred at room temperature for 24 h in dark conditions to reach equilibrium. The loaded MSN particles were collected by centrifugation and washed with ethanol. Ru-PIP-loaded MSNs were identified as Ru-PIP@MSNs. The concentration of the drug in the supernatant was determined from a calibration curve analyzed by a UV-Vis spectrophotometer at 458 nm. The drug loading content was calculated using the following equation:

(1) $$\%\;\mathrm{Drug}\;\mathrm{loaded}=\;\frac{\mathrm{Initial}\;\mathrm{drug}\;\mathrm{concentration}-\mathrm{drug}\;\mathrm{concentration}\;\mathrm{in}\;\mathrm{supernatant}\;}{\mathrm{Initial}\;\mathrm{drug}\;\mathrm{concentration}}\times 100$$

In this experiment, the release studies were done in phosphate buffer saline (PBS) at pH 5 and 7. Ru-PIP@MSNs (10 mg) were dispersed in 10 mL of PBS/ethanol (7:3). The dispersions were placed on a shaker on the lowest speed at room temperature under dark conditions. At a pre-determined time interval, 1 mL of the sample was collected and centrifuged. The supernatant was procured for UV-Vis analysis, while the particles were returned into the dispersion. The displaced solution was immediately replaced with 1 mL of fresh PBS solution in order to maintain a constant release volume. The amount of drug release was measured with a UV-Vis spectrophotometer, and the following is the formula used to calculate the amount of drug release at each time intervals.

(2) $$\%\;\mathrm{Drug}\;\mathrm{released}=\frac{\mathrm{Concentration}\;\mathrm{of}\;\mathrm{loaded}\;\mathrm{drug}\;-\mathrm{conc}.\;\mathrm{of}\;\mathrm{drug}\;\mathrm{at}\;\mathrm{time}\;t}{\mathrm{Concentration}\;\mathrm{of}\;\mathrm{loaded}\;\mathrm{drug}}\times 100\%$$

### 2.5. Cytotoxicity Studies

The IC_50_ values were determined through an MTT ([3-(4,5-dimethylthiazol-2-yl)-2,5-dephenyltetrazolium bromide]) assay. The final concentration of DMSO in all treatments was <0.5%. Cells were seeded at 1 × 10^4^ cells/well in 96-well plates for 24 h. The cells were treated with MSN (0–500 µg), Ru-PIP (0–100 µM), and Ru-PIP@MSNs (0–100 µM) for 24, 48, and 72 h each. Following treatment, the media were removed from each well and replaced by MTT solution (0.1 mg/mL), and then incubated for another 4 h. The purple formazan crystals formed were solubilized with 100 µL of DMSO, and the absorbance at 570 nm was determined using a microplate reader at 570 nm (Bio-Rad, Hercules, CA, USA). The IC_50_ values were determined based on the variation of percent cell viability data using GraphPad Prism 8.0 software (La Jolla, CA, USA).

### 2.6. Cell Cycle Analysis

Cells were seeded at 1 × 10^5^ cells/well in six-well plates for 24 h. After treating the cells with Ru-PIP (20 µM) and Ru-PIP@MSNs (20 µM) for 24 h, the cells were trypsinized, washed twice with PBS, and fixed with 70% ethanol at 4 °C for at least 12 h. Subsequently, the fixed cells were centrifuged and washed with PBS twice. The cell pellets were resuspended in 500 µL of PBS and treated with 50 µL RNase A (1 mg/mL), followed by 15 min of incubation. The samples were then stained with 20 µl of propidium iodide (500 µg/mL). The acquired samples were analyzed with Novocyte flow cytometer (ACEA Biosciences, CA, USA) and NovoExpress software, with a minimum count of 1 × 10^4^ cells.

## 3. Results

### 3.1. Physical Characterization

In the synthesis of mesoporous silica nanoparticles (MSNs), TEA was used to provide a basic pH and as a complexation agent for the silica precursor. Hydrolysis occurred upon introduction of a silica precursor into the aqueous reaction [32], followed by interaction of the negatively charged silica species with the cationic phenanthrolinium surfactant in the aqueous medium, consequently forming silica spheres. The products obtained after removal of the template were in the form of fine white powder.

The order of meso-structure was measured with powder x-ray diffraction (XRD) analysis. Typical MCM-41 would often exhibit pronounced peaks at a lower angle, which indicates high structural order [33]. The MSNs exhibited no peaks in the small angle region, indicating an amorphous nature with no specific pore ordering [34]. Further XRD analysis at the higher, 2θ range (Figure S2) displayed a broad peak around 18°–28°, corroborating the amorphous nature of the nanoparticles [24,35].

**Figure 2:**
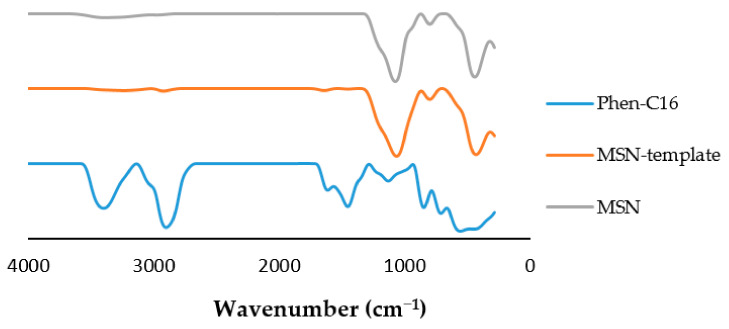
FTIR spectra of the synthesized template and mesoporous silica nanoparticles (MSNs) before and after removal of template.

FTIR analysis was used to examine structural transformations of the templated silica nanoparticles before and after removal of template Phen-C_16_. Figure 2 represents the FTIR spectra (in the 200–4000 cm^−1^ range) of Phen-C_16_, MSNs with Phen-C16 within the mesopores, and bare MSNs. The expected band of sp^2^ C–H stretch of the template at 2917 cm^−1^ and the absorption decreased as it was surrounded by the silica framework. A similar trend was observed for the C=C stretch at 1610 cm^−1^ and C=N stretch 1448 cm^−1^. The FTIR peaks for the mesoporous silica nanoparticles attributed to bands of Si–O–Si asymmetric stretching, Si–O–Si asymmetric stretching, and Si–O–Si bending were observed at 1063 cm^−1^, 1448 cm^−1^, and 432 cm^−1^, respectively. The absence of peaks of the functional groups related to the traces of template in the MSN showed the removal of template was successful.

### 3.2. Box–Behnken Design Surface Response

**Figure 3:**
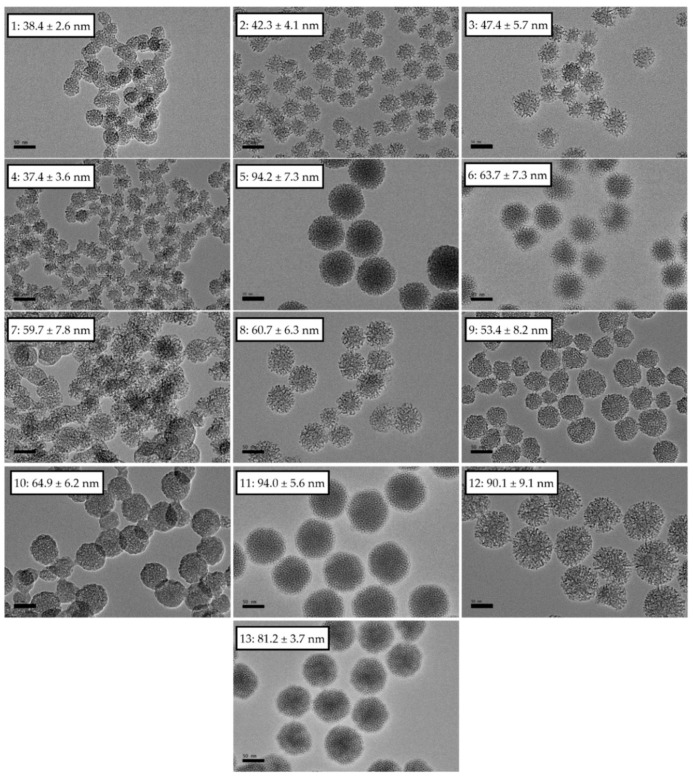
Transmission electron microscopy (TEM) images of synthesized MSNs corresponding to runs (Table 3).

MSNs synthesized according to the parameters proposed from the Box–Behnken design runs had diameters ranging from 30 nm to 100 nm, as determined from transmission electron microscopy (TEM) observation (Figure 3). From the micrographs, it was observed that most of the particles adopted spherical bodies with worm-like channels as pores, with no specific arrangements.

In order to investigate the effects of reaction temperature, mass of the template, and amount of TEA on the surface area of mesoporous silica nanoparticles, a Box–Behnken design with RSM was used (Table 1). The Box–Behnken design proposed a model to describe the relationships derived from the interaction effects, with which we can predict and control the surface area of particles under different conditions. By using the response surface, the influencing tendency of each factor was explored, and the optimal conditions for the synthesis subsequently determined.

Table 2 displays the statistical summary for each model that was output by Design Expert software version 7.1.6. A two-factor interaction (2FI) model was suggested, even though it has higher p-value compared to the linear model. The linear model values for R^2^ and adjusted R^2^ (Adj-R^2^) were 0.8442 and 0.7923, respectively, which is distinctly inadequate for experimental data. Therefore, the two-factor interaction (2FI) model was selected to fit the experimental data.

Experiments were performed using the parameters generated from the Box–Behnken design. The proposed Box–Behnken design required 13 runs to model the surface area response. The predicted and experimental responses, along with the reaction conditions, are tabulated in Table 3. The predicted response values were observed to agree with the experimental values.

The full terms for the 2FI model consisted of A, B, C, AB, AC, and BC. The model terms that are considered by the ANOVA to be significant must possess a large F-value and low probability value (*p*-value). A *p*-value lower than 0.05 indicates that the model is statistically significant, whereas a value higher than 0.1000 indicates that the model is not significant. The analysis of variance (ANOVA) of the model provided high F-values (27.85) along with low *p*-values (0.0004). The values of the involved terms are tabulated in Table 4. In this case, the significant terms were reduced to A, B, C, and AC, indicating that all linear terms along with AC interaction have a significant effect on the surface area of MSNs. The coefficient correlation, R^2^, is defined as the ratio of the explained variation to the total variation and a measurement of the degree of fit. A good model fit should yield an R^2^ of at least 0.8 [35,36]. The R^2^ of the model at a 95% confidence level was 0.9653, while the predicted R^2^ of 0.8087 was in reasonable agreement with the adjusted R^2^ of 0.9307. The R^2^ value is close to 1, which suggests that the response model evaluated in this study can explain the reaction with a high level of accuracy.

Based on the two-factor interaction (2FI) model chosen to fit the data, the relationship between the surface area and the three chosen factors is shown in Equation (3).

Surface area = 658 − 32.92A + 42.48B − 138.18C + 40.05AB − 61.81AC + 29.76BC(3)

where A, B, and C are the linear terms of mass of template (g), amount of TEA (g), and reaction temperature (°C), respectively, while AB, AC, and BC are the interaction terms.

**Figure 4:**
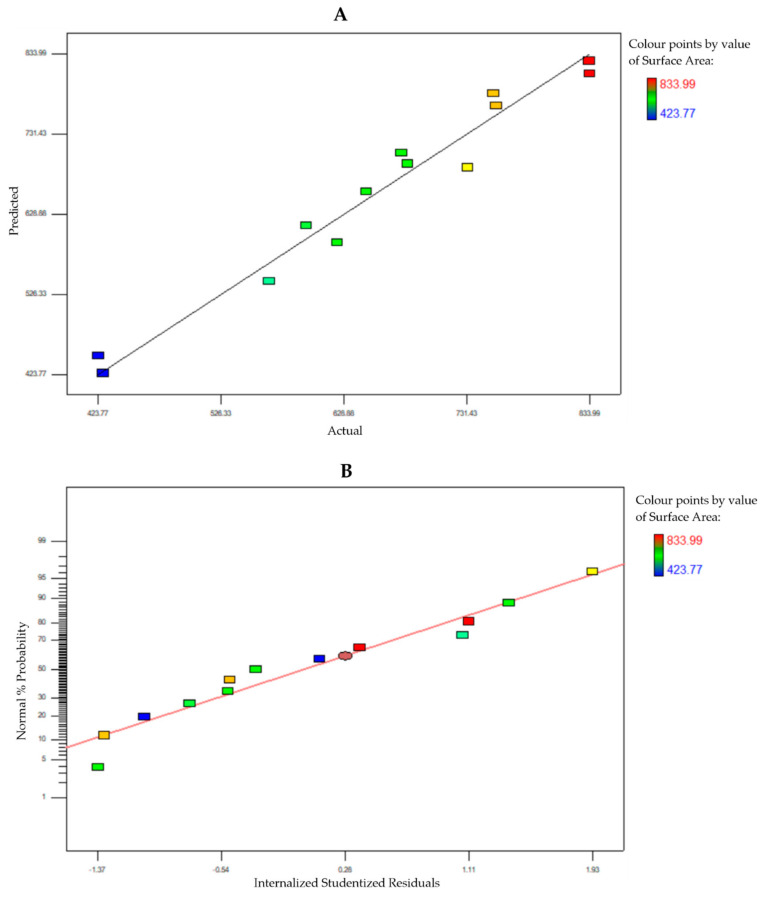
(**A**) Comparison of predicted and experimental surface areas. (**B**) Normal plot of residuals.

In order to check the model’s accuracy, the predicted and experimental surface areas were compared. Figure 4A displays the linear relationship between the predicted and experimental surface areas. In addition, the normal plot of residuals between the percentage of normal probability and the internally studentized residuals were obtained. The internally studentized residuals were used to measure the standard deviations separating the experimental and predicted values. Figure 4B shows the percentage or normal probability and the internally studentized residuals. The straight line indicates that no response transformation was required, and there was no problem with normality. Both plots determined that the model was adequate and satisfied the ANOVA assumptions.

**Figure 5:**
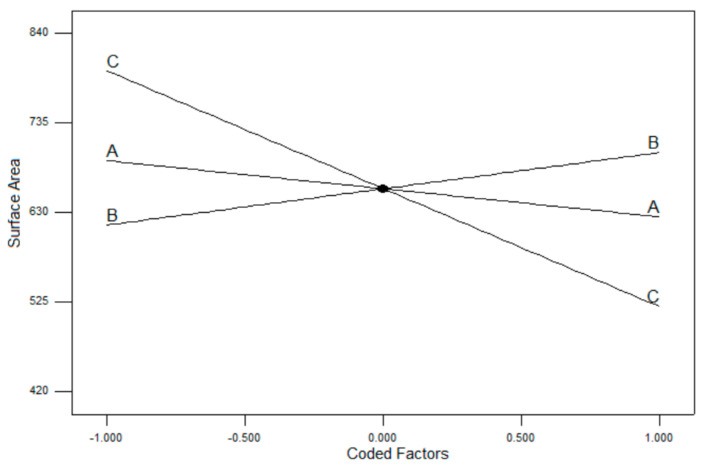
One-factor plot of coded components on the surface area. A: mass of template; B: amount of triethanolamine (TEA); C: reaction temperature.

At a high temperature, the reaction is subjugated by a growth process that produces MSNs with a smaller surface area. Meanwhile, the reaction at a low temperature is dominated by nucleation, where the proliferation of nuclei would produce smaller particles, hence increasing the total value surface area. The single factor plots of each component in Figure 5 show that reaction temperature was the parameter that influenced the surface of the MSNs to the greatest extent. The mass of the template is the second manipulated factor that gives changes to the surface area of MSN, albeit only slightly. In a reaction where the mass of template was increased, the surface area of the particles was reduced. This might be due to having insufficient silicate oligomers to form the required network around the much more concentrated surfactant micelles. The surface area was also affected by the amount of TEA, which generated MSNs with greater surface area at higher concentrations. TEA prevented the MSN from clumping together, which improved the surfaces of individual particles, providing a larger total surface area of the MSNs.

### 3.3. Drug Delivery

**Figure 6:**
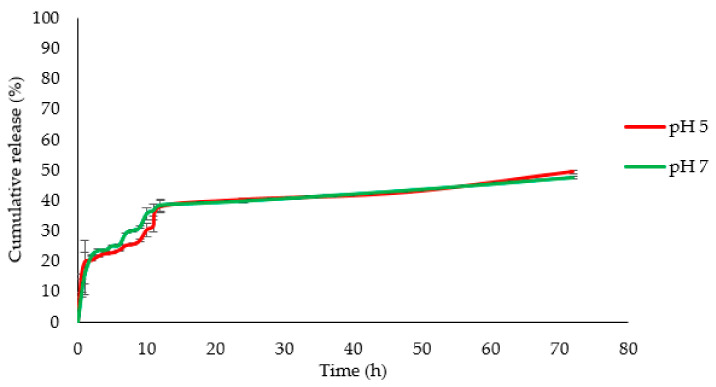
Cumulative release of Ru-PIP from MSNs.

The optimized MSNs were used for in vitro evaluation of the delivery of Ru-PIP ([Ru(dppz)_2_PIP]^2+^). Ru-PIP was loaded into the pores of MSN through the absorption method. The loading percentage of Ru-PIP from a single concentration in the MSN determined from UV analysis was 18.84% ± 0.1%. The in vitro drug release of Ru-PIP@MSNs was monitored in neutral and acidic conditions for 72 h. Ru-PIP had a burst release in the initial first hour, with a decreased rate from 1–11 h (Figure 6). A gradual release 11–72 h was then seen. A slightly reduced rate of release was observed from 1–11 h at pH 5 compared to neutral pH, and the cumulative release at 72 h of Ru-PIP in pH 5 and pH 7 were 49.31% and 47.51%, respectively. Although the release rate at 72 h was slow, the increment that was observable indicated that MSNs support a continuous release of Ru-PIP, even after 72 h.

In order to study the drug release kinetics, the experimental data were fit to five kinetic models, including zero-order, first-order, Higuchi, Hixson–Crowell, and Korsmeyer–Peppas equations. From the fitting of Ru-PIP release profile (Table 5) at pH 5, the Korsmeyer–Peppas model exhibits superiority, with *R*^2^ = 0.946, compared to the other models with *R*^2^ < 0.9. This indicates that an acidic environment induced a diffusion-controlled release mechanism for Ru-PIP from MSN [37]. Meanwhile, the release data for pH 7 were best fit to a first-order release profile, with *R*^2^ = 0.952; this usually means that the release was dependent on concentration. However, the release profiles that followed closely were Hixon–Cromwell, zero-order, and Higuchi, exhibiting similar trends of release profile with *R*^2^ > 0.92, signifying that the rate of release was independent of drug concentration and that the drug was released from the carrier by diffusion, subsequently changing the surface area of the particles and diameter of particles [31].

### 3.4. Cytotoxicity of Ru-PIP and Ru-PIP@MSNs

Ru-PIP has been shown to have anticancer activity; however, only a moderate toxicity towards the cancer cell lines tested was observed [25,26]. Thus, in order to enhance the cytotoxicity of Ru-PIP without modifying the compound itself, MSNs were utilized as a drug carrier for Ru-PIP. It is crucial for the toxicity of any nanoparticle to be evaluated in order to be an ideal drug delivery carrier. Therefore, the cytotoxicity effect of the carrier alone was also evaluated along with Ru-PIP, by using an MTT assay. The MTT assay was performed by using normal fibroblast cell line NHDF and several cancer cell lines, including cervical (Hela), lung (A549), and bladder (T24) cancers.

**Figure 7:**
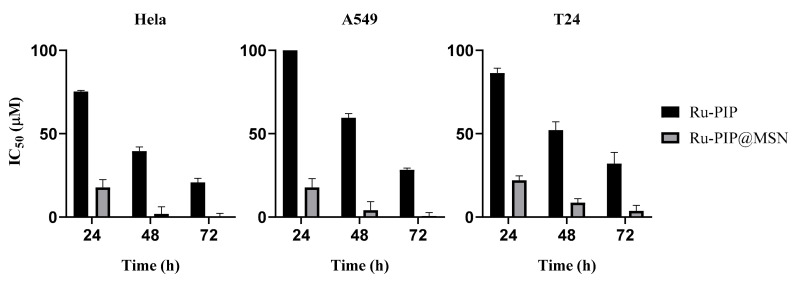
Graphical distribution of cytotoxicity (half-inhibitory IC_50_ concentrations) of Ru-PIP and Ru-PIP@MSNs towards Hela, A549, and T24c cell lines.

Bare MSNs displayed negligible cytotoxicity (half inhibitory concentrations, IC_50_ > 500 µg/mL) on all cancer and normal cell lines tested for 24, 48, or 72 h treatment (Figure S5). Treatment of the cancer cell lines with Ru-PIP resulted in mild cytotoxicity, with calculated IC_50_ values of >70 µM for the 24 h exposure, decreasing to 20–30 µM for 72 h exposure (Figure 7 and Table S1). These values were substantially reduced in the treatment with Ru-PIP@MSNs, even in the first 24 h. For example, in the 24 h treatment of A549, the IC_50_ value decreased from >100 µM to 17.78 µM for free complex and MSN delivery, respectively. Incubation of Hela and T24 cells followed a similar trend for all 24, 48, and 72 h treatment conditions. Considering the negligible cytotoxicity of bare MSNs, this implies that the improved cytotoxicity of Ru-PIP on these cell lines was assisted by MSN delivery. Meanwhile, Ru-PIP treatment of NHDF resulted in IC_50_ > 200 µM (Figure S4) for all incubation times, indicating that Ru-PIP possessed low toxicity towards these normal cells.

### 3.5. Cell Cycle Distribution

**Figure 8:**
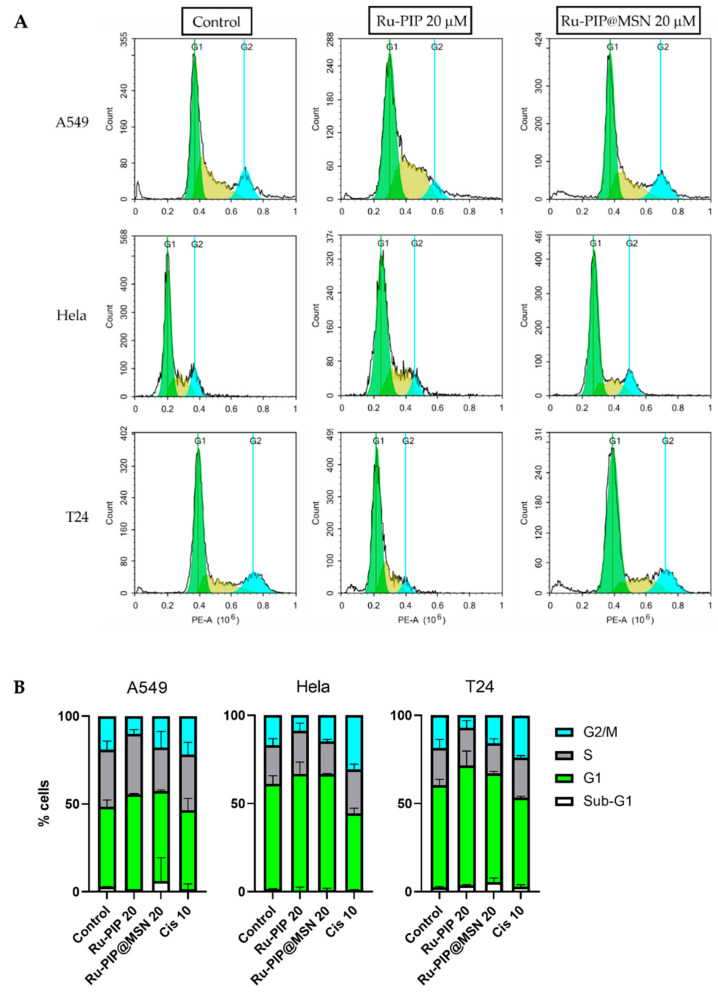
Cell cycle analysis of A549, Hela, and T24 cells. (**A**) DNA histograms depict the cell cycle distribution of Ru-PIP (20 µM) and Ru-PIP@MSN (20 µM) treatments compared to control cells. (**B**) Quantification of cell cycle distribution. Treatment with cisplatin (10 µM; Cis 10) are included for comparison.

The impact of Ru-PIP and Ru-PIP@MSNs on cell cycle progression was analyzed using flow cytometric analysis. Each cell line was treated with Ru-PIP (20 µM) and Ru-PIP@MSNs (20 µM) for 24 h. Compared to the controls (0.1% DMSO treated cells), treatment with free Ru-PIP at a concentration of 20 µM showed notable increases in cell populations in G1/S, with corresponding decreases in G2/M phases (Figure 8). These findings are consistent with previously reported results of Ru-PIP by Gill [25] and Yusoh [26], where Ru-PIP acts to stall DNA replication forks, leading to the activation of the checkpoint kinase 1 (Chk1) pathway and G1/early S cell cycle arrest [25].

In the case of MSN-delivered Ru-PIP, cells were treated with sub-IC_50_ concentrations of Ru-PIP (20 µM, labeled as “Ru-PIP@MSN 20” in Figure 8B). In response to this treatment, A549, Hela, and T24 cells showed similar increases in cell accumulation in G1 phase compared to control. Meanwhile, only a small arrest at the sub-G1 phase was observed for A549 and T24. From the DNA content histogram, the changes in peaks in the cell cycle distribution for free Ru-PIP was obvious, whereas the peaks for Ru-PIP@MSNs more closely resemble the control cells, with a slight broadening of the G1 population.

## 4. Discussion

As the drug of interest in this study was a ruthenium polypyridyl complex, [Ru(dppz)_2_PIP]^2+^, instead of using a typical surfactant cethyl trimethyl ammonium bromide (CTAB), this study utilized a similar salt based on the ligand in the complex, with the prospect that the particles would serve as a suitable carrier for the drug. Mesoporous silica nanoparticles (MSNs) were synthesized with phenanthrolinium salt with a 16-carbon chain length and Phen-C_16_ as the template. Nevertheless, after the removal of the template from the nanoparticle pore and surface, the physical characteristics of the silica nanoparticles were comparable to CTAB. Observation from TEM images showed that the particles were of nano-sized range and spherical in shape, with worm-like mesopores going through the particles.

The parameters proposed in the Box–Behnken design generated 13 synthesized conditions. The response of the predicted model and experimental evaluation were within reasonable agreement. Among the three factors investigated, the concentration of the template used in the reaction had the least significance to the changes in surface area, given that the concentration of the silica source (TEOS) that reacted with the template is still the same. However, the presence of excess surfactants might form a mono-layer on the silica surface, exposing its hydrophobic alkyl chains that can lead to aggregation of the particles [38]. The particles undertake size reduction at lower temperature reaction, consequently accumulating a larger surface area. We also observed that the concentration of additive TEA substantially influenced the response. Given that TEA acts in a dual function capacity, as both a capping agent and a basic catalyst, the capability in both functions would also increase as the concentration of TEA increased. During the reaction, TEA provided strong nucleophilic hydroxyl ions (-OH) to attack the silicon atom and catalyze the nuclei progression [32], forming smaller particles and acting as a capping agent, and thus refining the surface of each particle by inhibiting coagulation. The optimum condition reaction occurred at 65 °C with excess TEA additive, and a balanced amount of template as surfactant to react with the silica precursor produced MSNs with surface areas as large as 833.99 m^2^g^−1^.

From the drug loading process, the percentage of Ru-PIP entrapped in the MSNs was modest. The single concentration of Ru-PIP adapted in this method might have been too concentrated for the amount of MSN employed. The loading percentage could be optimized in future studies by using different concentration ratios of Ru-PIP to MSN in order to increase encapsulation efficiency. Ru-PIP was released from MSNs at a slower rate in acidic nature compared to one with a neutral pH, and the release profile observed up to 72 h accumulates to only half of total content of Ru-PIP. It is possible that the small amount of MSN returned into the solution after centrifugation was not redispersed, thus impeding the agglomerated particles from uniformly releasing Ru-PIP into the media solution. Furthermore, the aqueous nature of the solution might contribute to the slow release of Ru-PIP in PBS, as Ru-PIP by itself is a hydrophobic compound. The drug release kinetic models revealed that MSN released Ru-PIP by diffusion, where the changes in the size or diameter of particles [31] correlated with the drug unloading from the pores and surface of MSN [32]. We note that a zero-order model is often preferred for controlled and sustained drug release, as the system can offer higher efficacy even with minimum dosage frequency [39].

Remarkably, despite the slow release of Ru-PIP based on the drug release profile, the cytotoxicity of the Ru-PIP@MSNs towards all three cancer cell lines improved significantly. In related studies employing Ru (II) compounds in MSNs for drug delivery, He et al. [4] reported that the prolonged release of a related Ru (II) drug from MSNs for up to 12 days were cumulatively 63%, while Ma et al. [40] reported approximately 50% drug release at 48 h. However, the study by Ma et al. also showed that the Ru (II) drug achieved a near complete release in cell lysate, indicating that Ru-loaded MSNs were highly bio-responsive under an intercellular environment to release the ruthenium drug cargo. It is therefore possible that Ru-PIP@MSN possesses similar characteristics, as the toxicity of Ru-PIP towards cancer cells were enhanced by MSN delivery, despite having low cumulative release in regular PBS/EtOH.

As observed from the quantification of cell cycle distribution, treatment of A549, Hela, and T24 with 20 µM of free and MSN-loaded Ru-PIP for 24 h showed an increase of the cell population in G1/S phase compared to the control, albeit with a reduced effect for Ru-PIP@MSN treatment compared to free Ru-PIP. Although the content of Ru-PIP in MSNs was calculated at 20 µM based on the loading capacity, the concentration of Ru-PIP present in the media will be significantly lower than this—a factor that would explain the lower percentage of cell arrest at G1/S compared to the treatment of unloaded Ru-PIP at 20 µM. Although Ru-PIP@MSN induced a slight increase in the sub-G1 phase in A549 and T24 cells compared to the control, according to Gill et al. [25], Ru-PIP alone does not trigger apoptosis. Instead, cell progression is predominantly inhibited by the activation of G1/S and intra-S damage checkpoints, which causes a delay in repairing DNA damage prior to DNA replication [41,42]. More detailed studies into DNA damage in response to Ru-PIP@MSN treatment would be required to examine whether a similar response is triggered.

This study indicates that MSNs are an ideal drug delivery system for Ru-PIP. A gradual increase of Ru-PIP concentration delivered in the system would reduce unsolicited toxicity from the sudden exposure to a highly concentrated drug and avoid drug degradation outside of cancer site. This would thus lower the chances of Ru-PIP affecting healthy cells and prevent undesirable side effects during treatment. In addition to the optimization of drug entrapment efficiency, future studies would include tests like motility (scratch) assays, apoptosis tests, and visualization of drug release in cells using fluorescence microscopy, in order to further validate the relevance of these results and explore the mechanism of Ru-PIP@MSN cytotoxicity in more detail. Furthermore, the drug delivery aspect could be further improved by functionalizing the external surface of the MSN carrier with a targeting agent.

## 5. Conclusions

Mesoporous silica nanoparticles were successfully synthesized by using a phenanthrolinium salt as template, an alternative to CTAB. The Box–Behnken design applied to optimize the reaction condition effectively produced a high surface area response. While only a moderate amount of Ru-PIP was successfully loaded into the MSN, further optimization was required to improve the drug loading efficiency. Although the drug release was satisfactory, extra precautions or modified steps could be taken to facilitate drug dissolution to achieve complete release of drug molecules from the carrier. Through in vitro experiments against various cancer cell lines, the encapsulated Ru-PIP were able to enhance cytotoxicity significantly compared to unloaded Ru-PIP. Our findings suggest that the utilization of MSN as drug delivery agent for Ru-PIP is ideal, as it provides a sustainable release of the drug for prolonged treatment.

## Figures and Tables

**Table 1:** Uncoded and coded levels of variables.

Variables	Symbol	Levels		
		−1	0	+1
Mass of template (g)	A	0.25	0.38	0.50
Amount of TEA (g)	B	0.06	0.08	1.00
Temperature (°C)	C	65	77.5	90

**Table 2:** Model summary statistics.

Model	R^2^	Adj-R^2^	F-Value	*p*-Value
Linear	0.8442	0.7923	16.250	0.0006
2FI	0.9653	0.9307	6.990	0.0220
Quadratic	0.9670	0.8680	0.050	0.9825

**Table 3:** Experimental and predicted surface area under different conditions. A: mass of template, B: amount of triethanolamine (TEA) & C: reaction temperature

Run	Coded Variables			Real Variables			Response (m^2^g^−^^1^)	
	A	B	C	A	B	C	Experimental	Predicted
1	0	−1	−1	0.38	0.06	65.00	753.61	783.556
2	0	+1	−1	0.38	0.10	65.00	833.99	809.008
3	−1	0	−1	0.25	0.08	65.00	756.34	767.387
4	+1	0	−1	0.50	0.08	65.00	833.71	825.177
5	−1	0	0	0.38	0.08	77.50	647.62	658.101
6	+1	+1	0	0.50	0.10	77.50	676.82	707.715
7	−1	+1	0	0.25	0.10	77.50	682.14	693.455
8	−1	−1	0	0.25	0.06	77.50	732.19	688.577
9	+1	−1	0	0.50	0.06	77.50	566.69	542.657
10	0	+1	+1	0.38	0.10	90.00	623.18	592.161
11	+1	0	+1	0.50	0.08	90.00	427.69	425.195
12	−1	0	+1	0.25	0.08	90.00	597.56	614.645
13	0	−1	+1	0.38	0.06	90.00	423.77	447.678

**Table 4:** ANOVA of the fitted two-factor interaction (2FI) model for surface area of MSNs.

Terms	F-Value	*p*-Value	Characteristic
Model	27.85	0.0004	Significant
A (mass of template)	7.20	0.0363	Significant
B (amount of TEA)	12.00	0.0134	Significant
C (reaction temperature)	126.95	<0.0001	Significant
AB	5.33	0.0604	Not significant
AC	12.70	0.0119	Significant
BC	2.94	0.1370	Not significant

**Table 5:** Model Fitting for Ru-PIP@MSN release in pH 5 and pH 7.

Sample	Correlation Coefficient of Model (R)				
	Zero-Order	First-Order	Higuchi	Hixson-Crowell	Korsmeyer-Peppas
pH 5	0.878	0.876	0.878	0.880	0.946
pH 7	0.927	0.952	0.927	0.946	0.842

## Data Availability

Data is contained within the article or supplementary material.

## Supplementary Materials

The following are available online at https://www.mdpi.com/1999-4923/13/2/150/s1: Figure S1: Chemical structure of Phen-C_16_; Figure S2: X-ray plot of MSNs; Figure S3: Brunauer-Emmett-Teller (BET) isotherm linear plot corresponds with Box–Behnken design runs; Figure S4: Cell inhibition of normal human dermal fibroblasts (NHDFs) following 24, 48, and 72 h treatment with Ru-PIP and Ru-PIP@MSNs; Figure S5: Cell inhibition of MSN on normal and cancer cells; and Table S1: IC_50_ values or Ru-PIP and Ru-PIP@MSNs on cancer cells.
