# Supplementary material for: Synthesis and Optimization of Mesoporous Silica Nanoparticles for Ruthenium Polypyridyl Drug Delivery
Source: Pharmaceutics. 2021 Jan 24;13(2):150. doi: 10.3390/pharmaceutics13020150 (PMC7910993; doi:10.3390/pharmaceutics13020150)
Supplement: Supplementary file 1 [file pharmaceutics-13-00150-s001.pdf]

pharmaceutics

Supplementary Materials: Synthesis and Optimization of Meso-porous Silica Nanoparticles for Ruthenium Polypyridyl Drug Delivery

Siti Norain Harun, Haslina Ahmad *, Hong Ngee Lim, Suet Lin Chia and Martin R. Gill

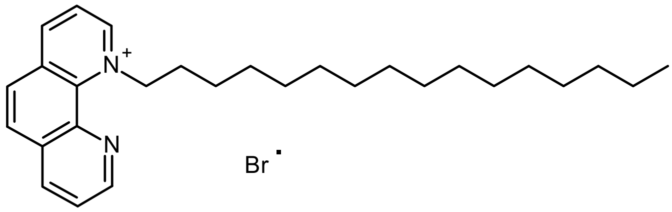

Figure S1: Chemical structure of Phen-C_{16}.

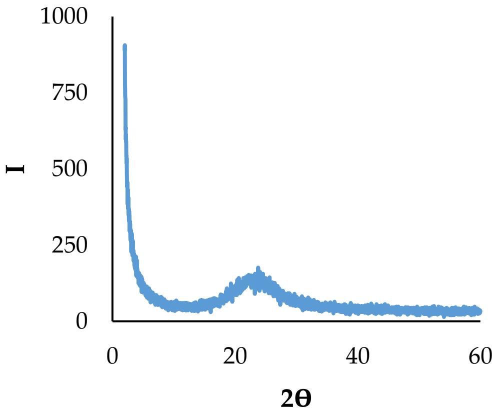

Figure S2: X-Ray (I vs 2θ) plot of MSN.

Publisher's Note: MDPI stays neutral with regard to jurisdictional claims in published maps and institutional affiliations.

Copyright: © 2020 by the authors. Submitted for possible open access publication under the terms and conditions of the Creative Commons Attribution (CC BY) license (http://creativecommons.org/licenses/by/4.0/).

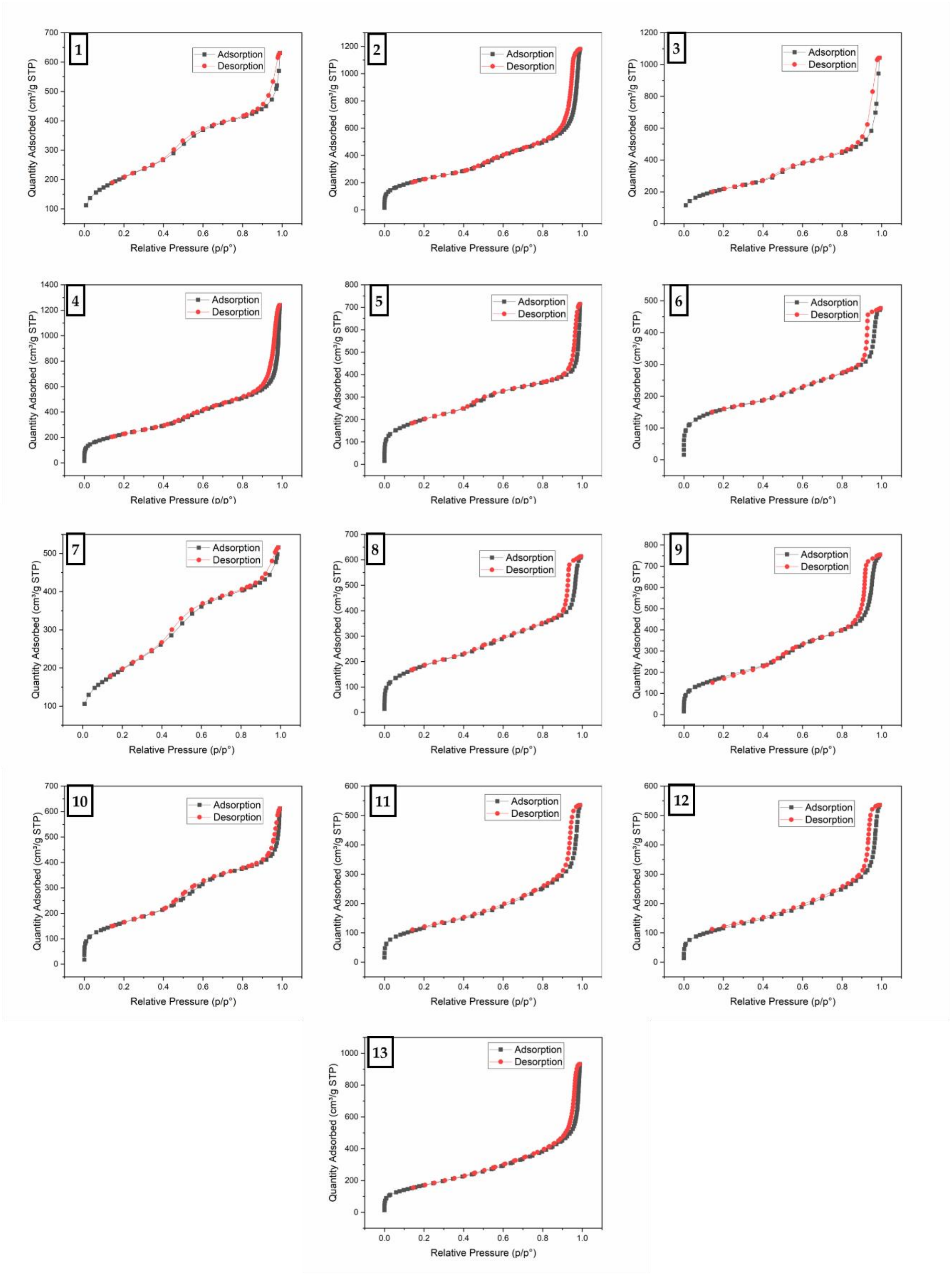

Figure S3. BET isotherm linear plot corresponds with Box Behnken design runs 1 to 13.

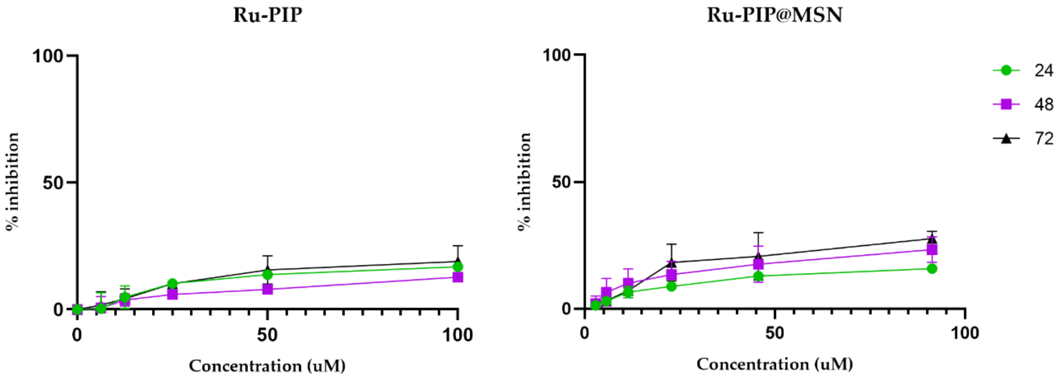

Figure S4. Cell inhibition on NHDF following 24, 48 and 72 h treatment of Rupip and MSN-Rupip.

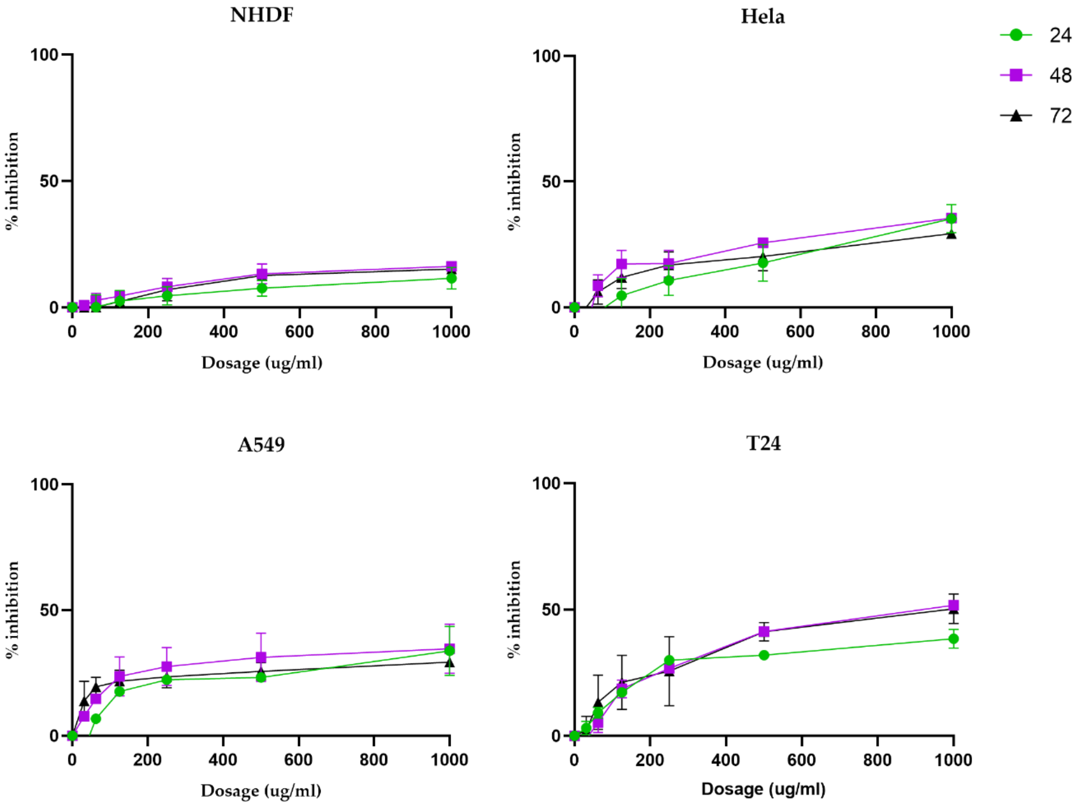

Figure S5. Cell inhibition of MSN on normal and cancer cells.

Table S1: IC_{50} values of Ru-PIP and Ru-PIP@MSN on cancer cells

Cells	Ru-PIP (μM)			Ru-PIP@MSN (μM)		
	24H	48H	72H	24H	48H	72H
Hela	75.30	39.51	20.82	17.82	1.84	0.36
A549	>100	59.53	28.29	17.78	4.15	0.63
T24	86.37	52.12	32.00	22.01	8.64	3.75
